# Dynamic Study of Intercalation/Deintercalation of Ionic Liquids in Multilayer Graphene Using an Alternating Current Raman Spectroscopy Technique

**DOI:** 10.1021/acs.jpclett.3c01686

**Published:** 2023-08-08

**Authors:** Zhi Cai, Haley Weinstein, Indu Aravind, Ruoxi Li, Sizhe Weng, Boxin Zhang, Jonathan L. Habif, Stephen B. Cronin

**Affiliations:** † Department of Physics and Astronomy, University of Southern California, Los Angeles, California 90089, United States; ‡ Department of Chemistry, University of Southern California, Los Angeles, California 90089, United States; § Ming Hsieh Department of Electrical Engineering, University of Southern California, Los Angeles, California 90089, United States; ∥ Mork Family Department of Chemical Engineering and Materials Science, University of Southern California, Los Angeles, California 90089, United States

## Abstract

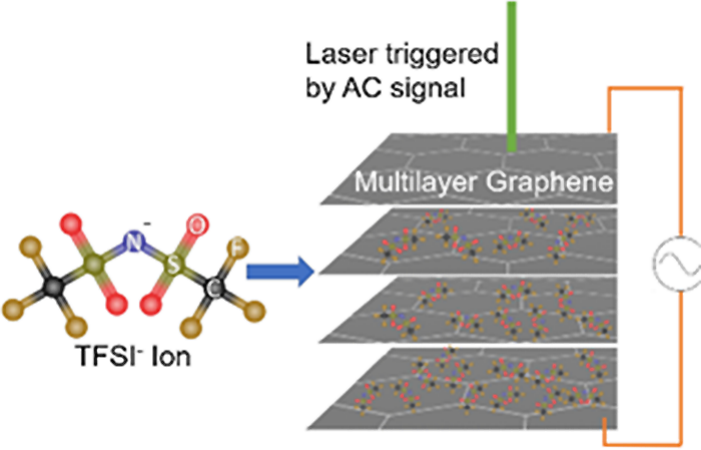

We report Raman spectra and infrared (IR) imaging collected during the intercalation–deintercalation half cycles in a multilayer graphene (MLG) device (∼100 layers) operating at 0.2–10 Hz. The device consists of a MLG/alumina membrane/copper stack, in which the alumina membrane is filled with ionic liquid [DEME][TFSI], forming an electrochemical cell. Upon the application of a positive voltage, the TFSI^–^ anions intercalate into the interstitial spaces in the MLG. The incident laser light is modulated using an optical chopper wheel that is synchronized with (and delayed with respect to) a 0.2–10 Hz alternating current (AC) voltage signal. Raman spectra taken just 200 ms apart show the emergence and disappearance of the intercalated G band mode at around 1610 cm^–1^. By integration of over hundreds of cycles, a significant Raman signal can be obtained. The intercalation/deintercalation is also monitored with thermal imaging via voltage-induced changes in the carrier density, complex dielectric function ε(ω), and thermal emissivity of the device.

The intercalation of small atoms (mostly alkali metals) into bulk graphite has been widely studied and is now utilized as the chief storage mechanism in Li-ion batteries.^1−4^ More recently, the electrochemically driven intercalation of ionic liquids containing relatively large ions (e.g., [DEME][TFSI]) has been demonstrated in multilayer graphene (MLG) grown by chemical vapor deposition (CVD).^5,6^ This MLG material/device system has shown great promise for broadband modulators, thermal camouflage, modifying thermal signatures, and covert communications,^7,8^ including a recent demonstration of 100 bits/s using ambient Planck radiation (i.e., blackbody).^5,9^ These applications exploit the widely tunable optical properties of graphene.^10,11^ One of the main differences between the CVD-grown MLG material and bulk graphite [i.e., highly oriented pyrolytic graphite (HOPG)] is that it consists of small grains of ∼5 μm, through which ion diffusion can occur, facilitating the cross-plane intercalation process. Figure S1 of the Supporting Information shows the grain structure of the MLG material, as observed under scanning electron microscopy (SEM) and atomic force microscopy (AFM).

Ionic liquids provide an electrochemical system that is considerably more stable than that of Li or water and stable in air over many cycles of charging and discharging. It is rather remarkable that these large ions (composed of roughly 10 atoms) can fit within the interstitial spaces in the van der Waals-bound lattice of the MLG material, and it is even more remarkable that this process can be driven at relatively high speeds. Raman spectroscopy is well-suited for studying the intercalation of MLG because its features contain important information about the material, including Fermi energy Δ*E*_F_, free carrier density *N*, and the formation of defects.^12^ Previously, infrared (IR) and Raman spectroscopy was carried out on these types of MLG devices under applied direct current (DC) voltages and collected on the time scale of several minutes.^13^ In the work presented here, we present a new strategy for collecting Raman spectra of this intercalation–deintercalation process on relatively short time scales.

There have been many studies investigating intercalation into bulk graphite, mostly focused on group I metal ions, including lithium,^14^ sodium,^15^ and potassium.^16−18^ In addition, several groups have investigated intercalation of other ions, including nitrates (from HNO_3_),^19^ large fluoroanions (including perfluoroalkylimides, perfluoroalkylsulfonates, and perfluoroalkylborate esters),^20^ and ionic liquids. These investigations have mainly looked at HOPG and have used a wide range of characterization techniques/methods, including the combination of Fourier transform infrared (FTIR) and Raman spectroscopy, by X-ray diffraction, thermogravimetry, and the above structural characterizing techniques.^21^ Kinetic studies of anionic intercalation/deintercalation into the graphite electrode have been studied using lithium ions solely based on electrochemical measurements (i.e., not structural measurements).^22^ As such, dynamic optical, vibrational, and structural techniques that can be performed *in situ* as a function of time are much needed. Raman scattering is particularly well-suited for these kinetic studies for the following reasons: (1) the strong Raman intensity of graphite, which originates from its strong electron–phonon (el–ph) coupling, (2) the ease of use of Raman spectroscopy, (3) the wide range of structural information that can be obtained from these Raman spectra, and (4) the ease with which optical probes can be extended into the time domain. For example, it would much more difficult to perform XRD or FTIR measurements in the time domain. Raman studies have been used extensively to study disorder, doping, and intercalation in graphite-based systems, including several reviews that have been cited more than 3400 times as of this writing.^23^

In the CVD growth of MLG, a 0.125 mm thick nickel foil (Sigma-Aldrich 7440-02-0) is placed inside a 1 in. diameter quartz tube and heated to a temperature of 1050 °C, as illustrated in Figure 1a. The growth occurs while 100 standard cubic centimeters per minute (sccm) of H_2_ and 30 sccm of CH_4_ flow through the tube under a pressure of 10 Torr for 5 min. Following the growth process, the nickel foil is etched in a solution of nickel etchant (nickel etchant type I, Transene Company, Inc.), resulting in the hydrophobic MLG floating on top of the etchant. After etching, the MLG is transferred to deionized (DI) water twice to remove residual ions from the etchant. Subsequently, the MLG is transferred onto an Al_2_O_3_ porous membrane (Sigma-Aldrich WHA68096002), as illustrated in panels b and c of Figure 1. Ionic liquid diethylmethyl(2-methoxyethyl)ammonium bis(trifluoromethylsulfonyl)imide ([DEME][TFSI]) is injected into the membrane from the back side before MLG/Al_2_O_3_ is deposited on the bottom copper electrode. The ionic liquid can be seen in Figure 1c as the milky white region.

**Figure 1:**
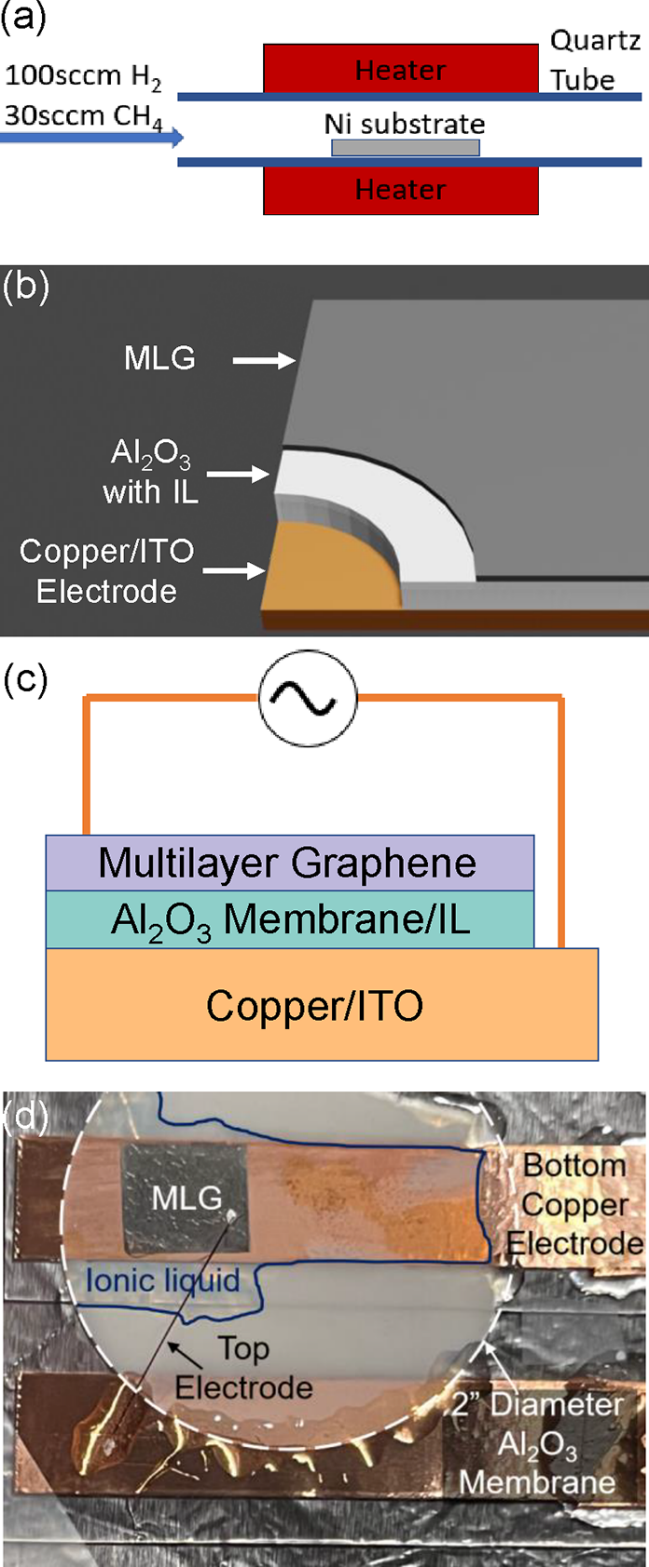
(a) Schematic diagram of the CVD setup for MLG growth on a Ni substrate and (b) drawing, (c) cross-sectional diagram, and (d) photograph of the sample consisting of a MLG top electrode, porous Al_2_O_3_ membrane filled with ionic liquid ([DEME][TFSI]), and copper bottom electrode.

Alternating current (AC) Raman spectroscopy is performed using a 532 nm wavelength laser that is modulated by an optical chopper wheel with a 1:3 (on/off) duty cycle, as illustrated schematically in Figure 2a. The modulated laser beam is then split using a 10:90 beam splitter. A portion of the modulated laser beam is sent to a photodiode that triggers the voltage pulses to the sample, while the remaining laser light is focused onto the sample using a high numerical aperture objective lens (50× NA = 0.6). The Raman scattered light is collected using a Renishaw *in via* micro-Raman spectrometer. The voltage pulse from the photodiode triggers an Arduino microprocessor, which is programmed to toggle a three-state multiplexer (MUX) between +5 V, −5 V, and open-circuit conditions. This voltage pulse sequence is plotted in Figure 2b. Here, intercalation takes place during the +5 V pulse, and deintercalation occurs during the −5 V pulse. Raman spectra are taken under open-circuit conditions (*R* = ∞) during which the intercalated charge is “frozen” or fixed. Figure 2c shows the laser intensity profiles that are incident on the sample. By adjusting the phase of the voltage pulse signal/sequence with respect to the optical trigger, we collect spectra when the sample is in either the intercalated or deintercalated state under open-circuit conditions. By integrating over hundreds of cycles (i.e., 2 min), we can collect a sufficient signal to resolve the subtle features in the Raman spectra of the MLG.

**Figure 2:**
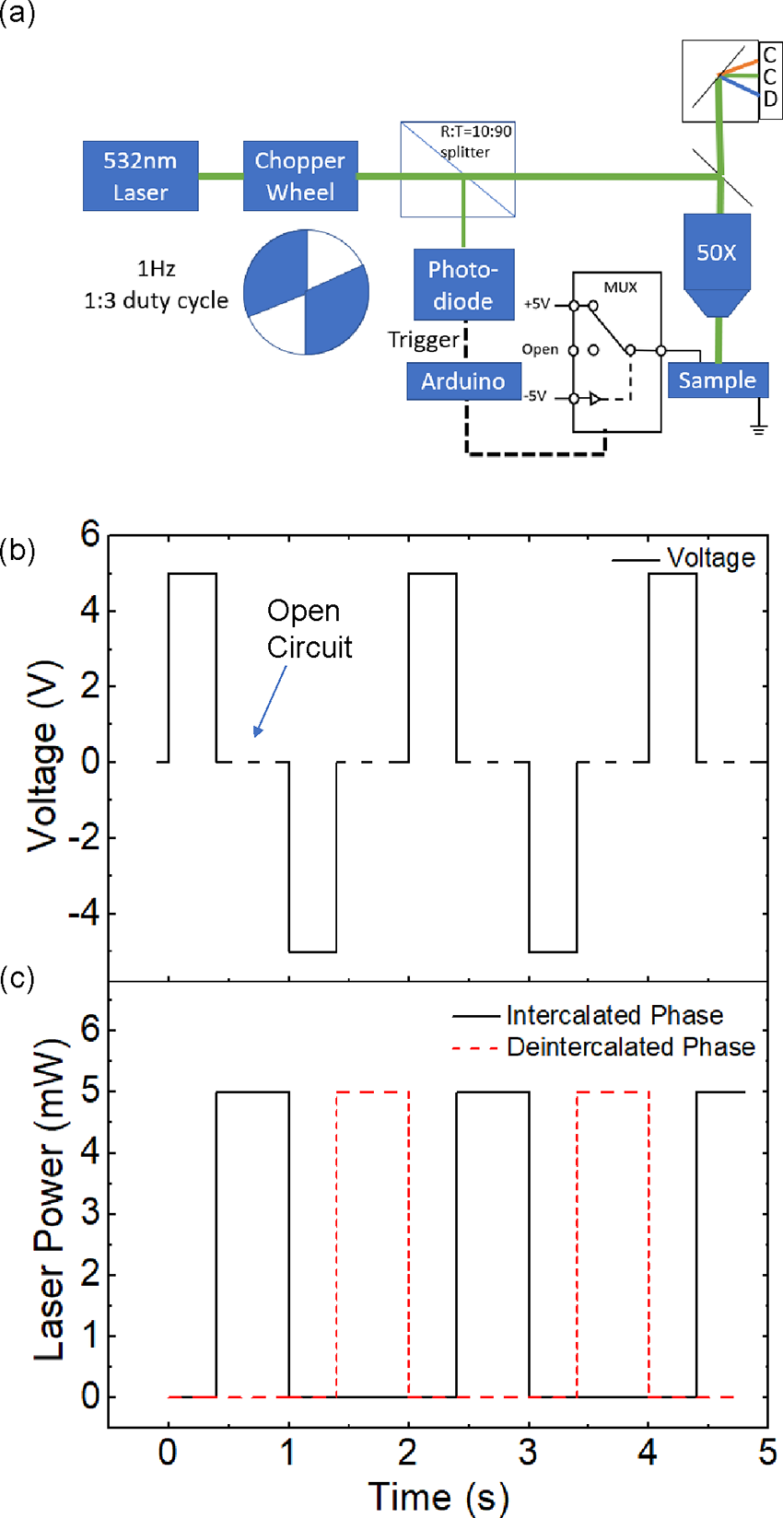
(a) Schematic diagram of the *in situ* AC Raman spectroscopy setup and (b) voltage pulse sequence and (c) laser pulse sequence for measuring the intercalated and deintercalated phases of the MLG electrode.

The D and G band Raman spectra taken in the intercalated and deintercalated states using the experimental setup described above are plotted in Figure 3a. Here, we see a more pronounced intercalated G band feature (*G*_int_) around 1610 cm^–1^ in the intercalated state and a larger neutral G band peak (*G*) in the deintercalated state around 1580 cm^–1^. We can estimate the amount of intercalated charge from the ratio of the *G*_int_/*G* band Raman intensities. Fully intercalated MLG has a carrier density of *N* = 2 × 10^21^ cm^3^. In the intercalated state, we observe a *G*_int_/*G* ratio equal to 2:1, corresponding to *N* ≈ 5 × 10^20^ cm^–3^, while in the deintercalated state, we observe a *G*_int_/*G* ratio equal to 1:2, corresponding to *N* ≈ 2 × 10^20^ cm^–3^. Thus, the net change in charge is Δ*N* = 3 × 10^20^ cm^–3^ during each intercalation/deintercalation voltage cycle. That is, under the AC steady-state conditions, the sample does not fully intercalate or deintercalate. Also, the D band is slightly more pronounced during the intercalated phase. AC Raman spectra were also collected from the bottom side of the MLG electrode, as illustrated in Figure S2 of the Supporting Information. Here, instead of using a copper bottom electrode, indium tin oxide (ITO)-coated glass is used as the bottom electrode and a glass correction (Olympus LUCPlanFL N 40×/0.60) lens is used to measure the Raman spectra. Here, the alumina membrane is crucial to obtaining a clear optical path to the MLG compared to previously used polymer-based membranes.^9,13^Figure 3b shows a series of Raman spectra taken from the bottom surface of the MLG electrode, which is in direct contact with the ionic liquid solution. We observe a *G*_int_/*G* ratio of 1:2 in the intercalated state, corresponding to *N* = 2 × 10^20^ cm^–3^. In the deintercalated state, *G*_int_ disappears, indicating that the bottom layers of the MLG are nearly fully deintercalated. Figure 3b also shows a peak at 1240 cm^–1^ that corresponds to the CF_3_ stretching mode of the ionic liquid.

**Figure 3:**
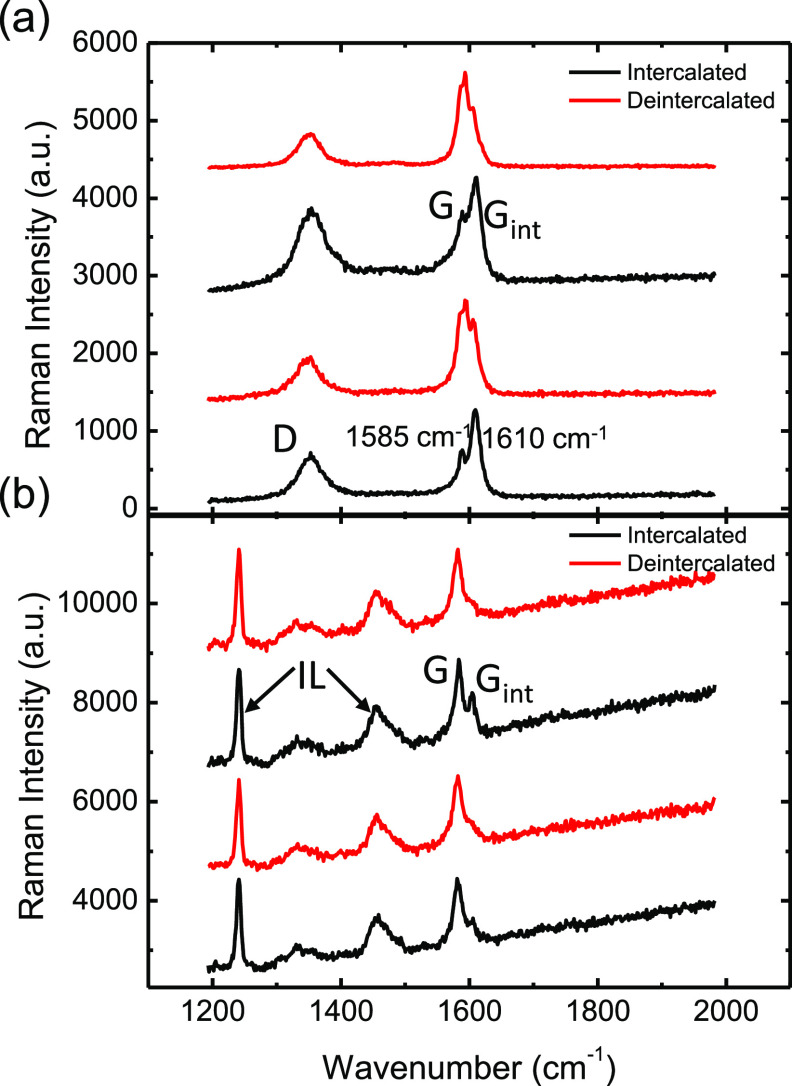
Raman spectra taken during the intercalation and deintercalation phases from the (a) top side and (b) bottom side of the MLG electrode.

In addition to Raman spectroscopy, we also performed time-resolved thermal imaging using a FLIR camera. Figure 4 shows the results of thermal imaging of the MLG/Al_2_O_3_ membrane/Cu device on a hot plate at 40 °C. Here, we observe changes in the apparent temperature of the device as a result of the voltage-induced changes in the thermal emissivity of the MLG material with 5 ms time resolution (200 Hz frame rate). In performing these measurements, we started by applying the voltage pulse sequence plotted in Figure 2b at a frequency of 10 Hz. Initially, we see that there is very little change in the temperature (i.e., emissivity) at 10 Hz (Figure 4b). Applying the same voltage profile at 0.2 Hz (Figure 4c), we observe a large change in the apparent temperature (i.e., emissivity) of Δ*T* > 10 °C. After this, the frequency response of the intercalation/deintercalation is much faster, showing a substantial Δ*T* ≈ 2 °C at 10 Hz, as plotted in Figure 4d. For these AC measurements, we find that modulating the device first at a low frequency (i.e., 0.2 Hz) is important for achieving better intercalation at higher frequencies (i.e., 10 Hz).

**Figure 4:**
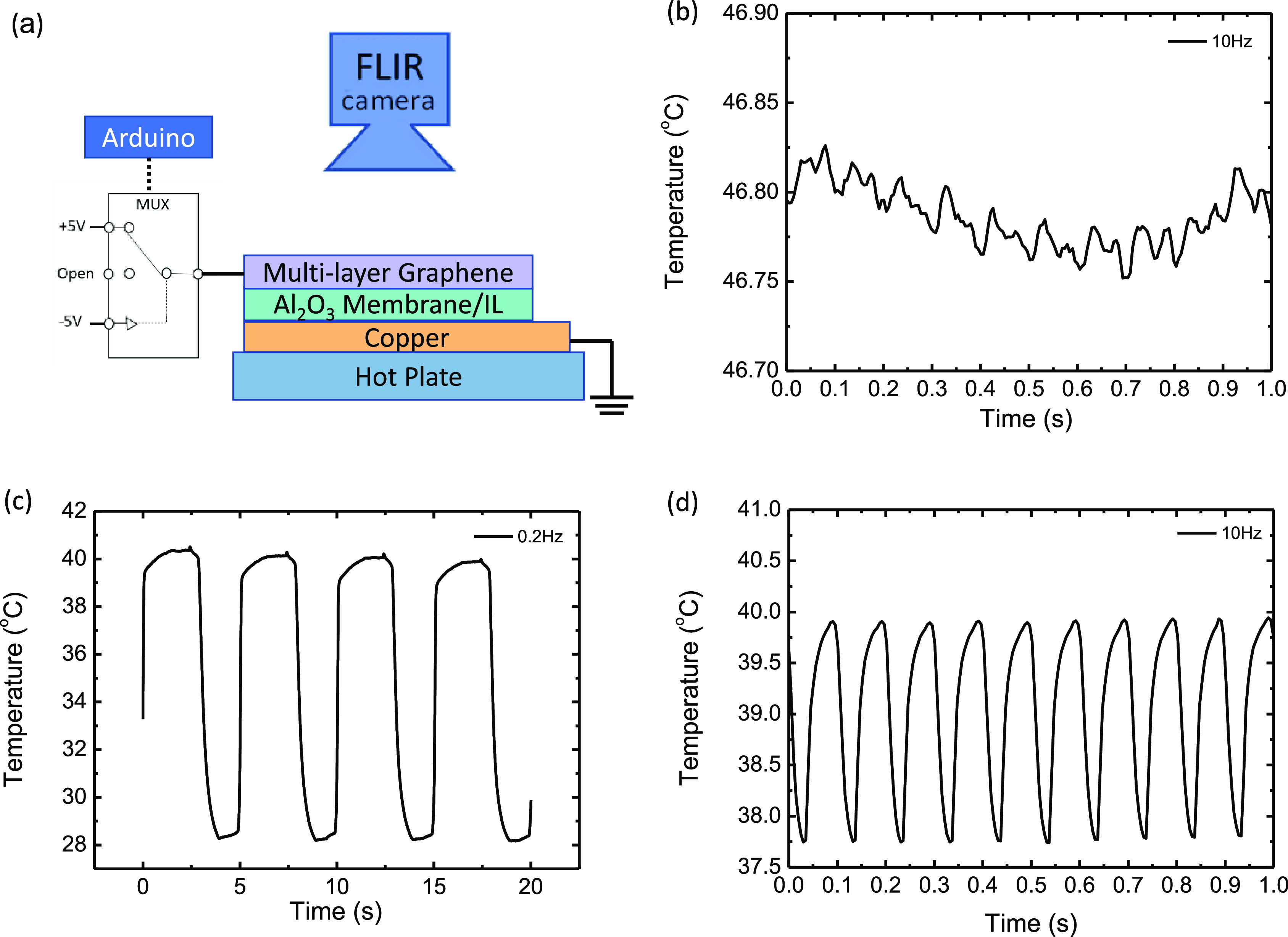
(a) Schematic diagram of the thermal imaging camera setup and (b–d) apparent temperature observed at 10, 0.2, and 10 Hz pulse voltage frequency, applied successively.

To provide a basic understanding of the intercalation-induced changes in the MLG material, we connect the thermal emissivity measurements with the Raman spectroscopy measurement results. As such, this partial intercalation/deintercalation can have a significant impact on the complex dielectric function of the material, which can be described using a Drude model with interband transitions as follows:^24,25^
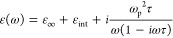
where
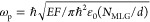
and *N*_MLG_ is the number of MLG layers and *d* is the thickness of MLG. The voltage-induced modulation of the dielectric function [Δε(ω)], in turn, affects in the thermal emissivity *E*(ω,*T*) of the material as follows:
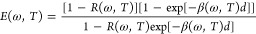
where
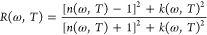
and
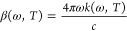
where *n*(ω,*T*) is the refractive index and *k*(ω,*T*) is the extinction coefficient. While the MLG does not fully deintercalate, Δ*N* = 3 × 10^20^ cm^–3^, which is substantial compared to the intrinsic carrier concentration of MLG of 3 × 10^18^ cm^–3^. On the basis of this, we predict a Δ*T* of 10 °C, which is consistent with our experimental observations.

Figure 5 shows a linear–log plot of the apparent temperature change as a function of the drive frequency. Here, we observe that the apparent temperature change increases from 0.25 °C at 10 Hz to 14 °C when the frequency decreases to 0.2 Hz, where the temperature change reaches maximum saturation. On the basis of these data, these devices exhibit a cutoff frequency of ∼1 Hz, although appreciable modulation can still be observed up to 10 Hz.

**Figure 5:**
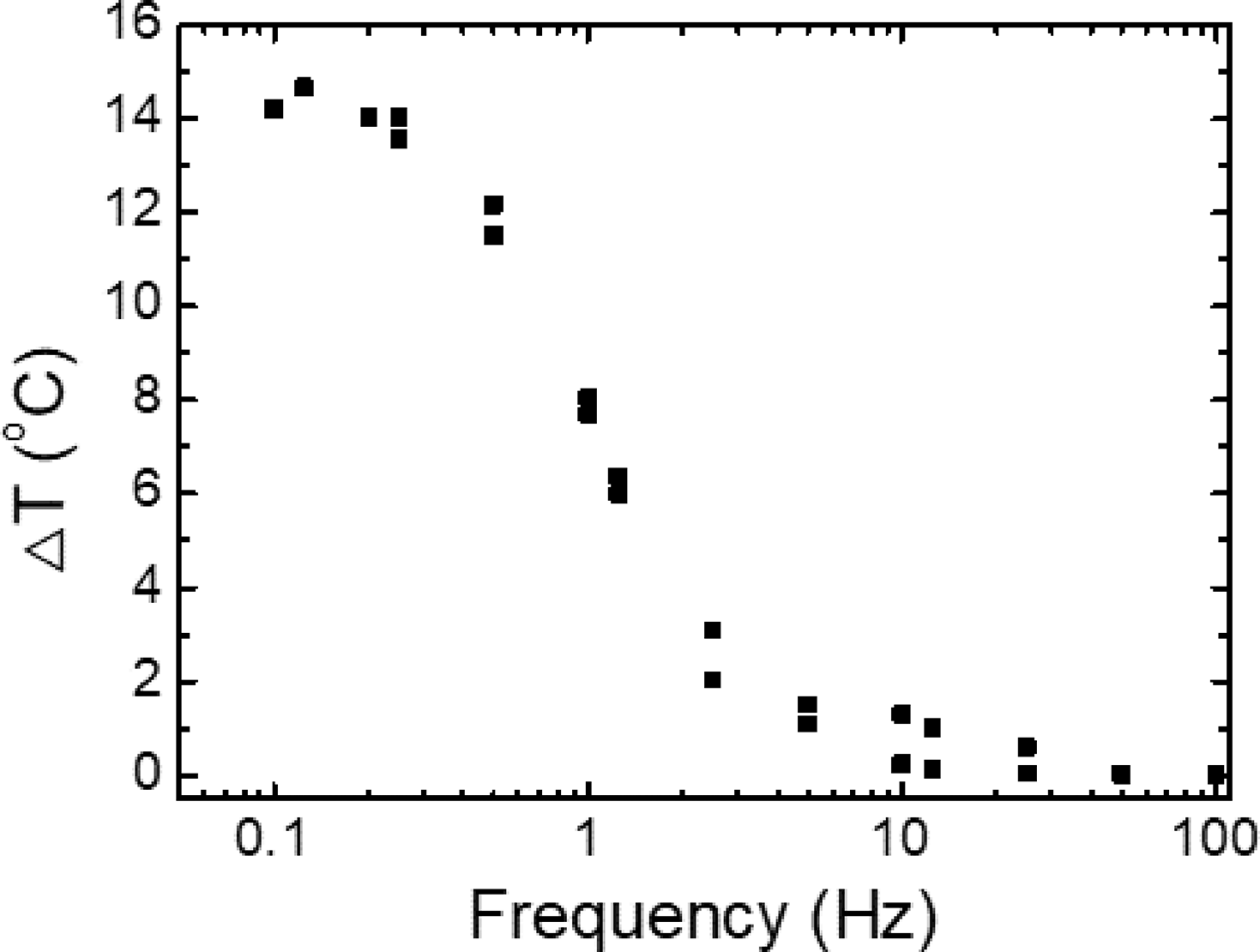
Apparent temperature change measured as a function of the frequency.

In conclusion, we have demonstrated a strategy for investigating the dynamic process of ionic liquid intercalation and deintercalation in MLG through the utilization of Raman spectroscopy combined with applied AC voltages. By analyzing the Raman spectra, we observe that changes in the spectra occur during the intercalation and deintercalation processes. One significant finding of our study is the notable difference in carrier density between the intercalated and deintercalated states, which is approximately 3 × 10^20^ cm^–3^. This substantial difference in carrier density results in a significant change in the thermal emissivity of the MLG, which gives rise to a significant change in the apparent temperature variation of approximately 10 °C. This apparent temperature change, caused by the carrier density difference, highlights the strong correlation between the intercalation/deintercalation processes and the dielectric function ε(ω) of the MLG material. We believe that this dynamic spectroscopy approach can be used to investigate a wide range of rechargeable battery electrodes *in situ*.

## Supporting Information Available

Additional experimental details including surface topography characterization and experimental setup (PDF)
