# Supplementary material for: Dynamic Study of Intercalation/Deintercalation of Ionic Liquids in Multilayer Graphene Using an Alternating Current Raman Spectroscopy Technique
Source: J Phys Chem Lett. 2023 Aug 8;14(32):7223–8. doi: 10.1021/acs.jpclett.3c01686 (PMC10440811; doi:10.1021/acs.jpclett.3c01686)
Supplement: Supplementary file 1 — jz3c01686_si_001.pdf [file jz3c01686_si_001.pdf]

Supporting Information for

A Dynamic Study of Intercalation/Deintercalation of Ionic Liquids in Multilayer Graphene using an AC Raman Spectroscopy Technique

Zhi Cai[4], Haley Weinstein[3], Indu Aravind[1], Ruoxi Li[4], Sizhe Weng[3], Boxin Zhang[4], Jonathan Habif[3],

and Stephen B. Cronin[1, 2, 3, a)]

[1]Department of Physics and Astronomy, [2]Department of Chemistry, [3]Ming Hsieh Department of Electrical Engineering, [4]Mork Family Department of Chemical Engineering and Materials Science, University of Southern California, Los Angeles, CA 90089, USA

[a)] Authors to whom correspondence should be addressed: scronin@usc.edu

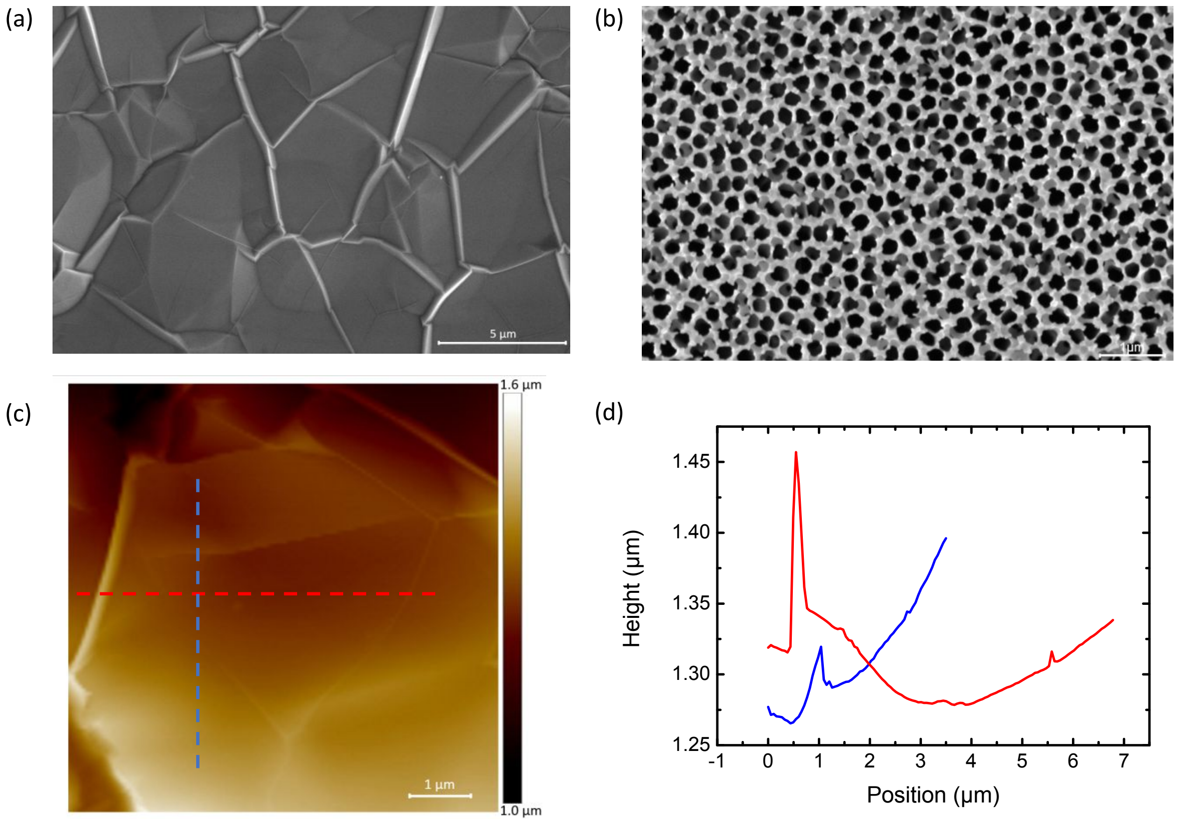

Figure S1. (a) and (b) SEM image of multilayer graphene and porous Al_2O_3 membrane. (c) and (d) AFM image of multilayer graphene and corresponding cross-sectional plot.

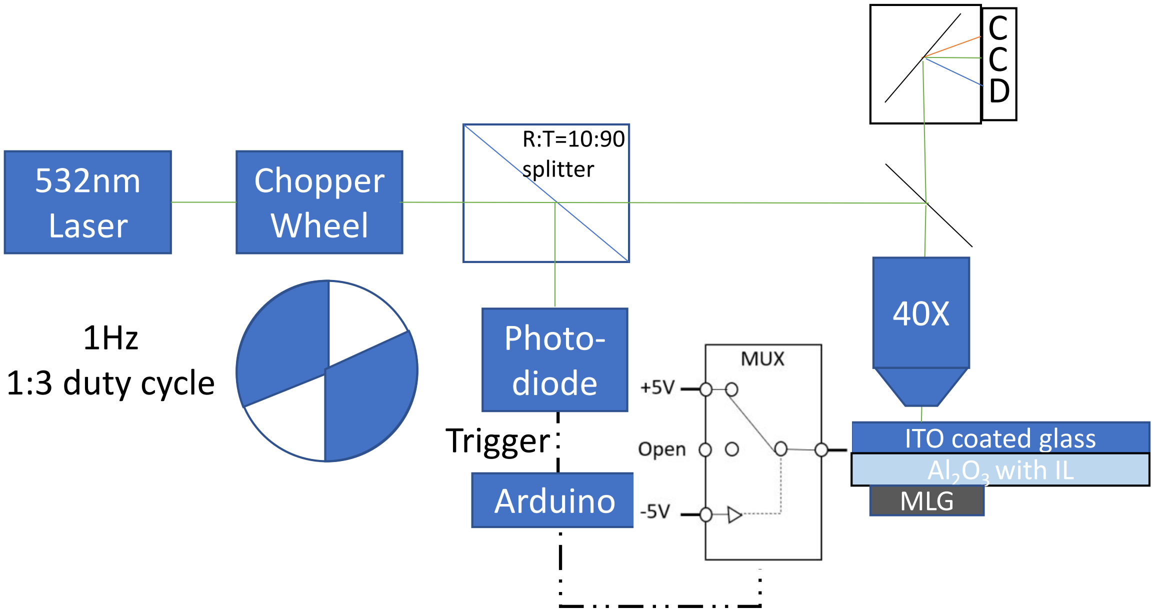

Figure S2. Schematic diagram of Raman setup of ITO bottom electrode sample measurement
